# Supplementary material for: Dependence of nucleosome mechanical stability on DNA mismatches
Source: eLife. 2024 Apr 24;13:RP95514. doi: 10.7554/eLife.95514 (PMC11042804; doi:10.7554/eLife.95514)
Supplement: Supplementary file 1. — Light gray shades denote sequences in the outer turn of the nucleosome and dark gray shares denote sequences in the inner turn of the nucleosome. Green and red ‘T’s denote the labeling sites for donor (Cy3) and acceptor (Cy5) fluorophores, conjugation done via amino-dT for the ED1 construct. For labeling sites for the ED2 construct, we refer to Li, 2008. Yellow highlights denote the positions of the mismatched bases. /idSp/ is the space added to prevent polymerization onto the 5’ overhang. [file elife-95514-supp1.docx]

**601 original sequence (made by PCR)**

5’- /Biotin/TATA CGCGGCCGCC CTGGAGAATC CCGGTGCCGA GGCCGCTCAA TTGGTCGTAG ACAGCTCTAG CACCGCTTAA ACGCACG**T**AC GCGCTGTCCC CCGCGTTTTA ACCGCCAAGG GGATTACTCC CTAGTCTCCA GGCACGTGTC AGATATATAC ATCCTGT GCATGTATTG AACAGCGACC

ATAT GCGCCGGCGG GACCTCTTAG GGCCACGGCT CCGGCGAGTT AACCAGCATC TGTCGAGATC GTGGCGAATT TGCGTGCATG CGCGACAGGG GGCGCAAAAT TGGCGGTTCC CCTAATGAGG GATCAGAGGT CCGTGCACAG TCTATATATG **T**AGGACA CGTACATAAC TTGTCGCTGG/idSp/ TC CAGCGGCGGG – 5’

**R18 construct**

5’- /Biotin/TATA CGCGGCCGCC CTGGAGAATC CCGGTGCCGA GGCCGCTCAA TTGGTCGTAG ACAGCTCTAG CACCGCTTAA ACGCACG**T**AC GCGCTGTCCC CCGCGTTTTA ACCGCCAAGG GGATTACTCC CTAGTCTCCA GGCACGTGTC AGATATATAC ATCCTGT GCATGTATTG AACAGCGACC

ATAT GCGCCGGCGG GACCTCTTAG GGCCACGGCT CCGGCGAGTT AACCAGCATC TGTCGAGATC GTGGCGAATT TGCGTGCATG CGCGACAGGG GGCGCAAAAT TGGCGGTTCC CCTAATGAGG GATCAGAGGT CCGTGCACAC TCTATATATG **T**AGGACA CGTACATAAC TTGTCGCTGG/idSp/ TC CAGCGGCGGG – 5’

**R39 construct**

5’- /Biotin/TATA CGCGGCCGCC CTGGAGAATC CCGGTGCCGA GGCCGCTCAA TTGGTCGTAG ACAGCTCTAG CACCGCTTAA ACGCACG**T**AC GCGCTGTCCC CCGCGTTTTA ACCGCCAAGG GGATTACTCC CTAGTCTCCA GGCACGTGTC AGATATATAC ATCCTGT GCATGTATTG AACAGCGACC

ATAT GCGCCGGCGG GACCTCTTAG GGCCACGGCT CCGGCGAGTT AACCAGCATC TGTCGAGATC GTGGCGAATT TGCGTGCATG CGCGACAGGG GGCGCAAAAT TGGCGGTTCC CCTAATGACG GATCAGAGGT CCGTGCACAG TCTATATATG **T**AGGACA CGTACATAAC TTGTCGCTGG/idSp/ TC CAGCGGCGGG – 5’

**R56 construct**

5’- /Biotin/TATA CGCGGCCGCC CTGGAGAATC CCGGTGCCGA GGCCGCTCAA TTGGTCGTAG ACAGCTCTAG CACCGCTTAA ACGCACG**T**AC GCGCTGTCCC CCGCGTTTTA ACCGCCAAGG GGATTACTCC CTAGTCTCCA GGCACGTGTC AGATATATAC ATCCTGT GCATGTATTG AACAGCGACC

ATAT GCGCCGGCGG GACCTCTTAG GGCCACGGCT CCGGCGAGTT AACCAGCATC TGTCGAGATC GTGGCGAATT TGCGTGCATG CGCGACAGGG GGCGCAAAAT TCGCGGTTCC CCTAATGAGG GATCAGAGGT CCGTGCACAG TCTATATATG TAGGACA CGTACATAAC TTGTCGCTGG/idSp/ TC CAGCGGCGGG – 5’

**Sequences for looping measurements – made by annealing of the top and bottom strands which was synthesized by IDT**. Yellow highlights denote the locations of mismatched bases. Cyan highlights denote the location of biotin conjugated via dT.

**601-RH**

5’- /5Cy5/ACGGATTCTG TGTCCC CCGCGTT/iBiodT/TA ACCGCCAAGG GGATTACTCC CTAGTCTCCA GGCACGTGTC AGATATATAC ATCCTGT

ACAGGG GGCGCAAA AT TGGCGGTTCC CCTAATGAGG GATCAGAGGT CCGTGCACAG TCTATATATG TAGGACA TGCCTAAGAC /5Cy3/ – 5’

**601-R18-RH**

5’- /5Cy5/ACGGATTCTG TGTCCC CCGCGTT/iBiodT/TA ACCGCCAAGG GGATTACTCC CTAGTCTCCA GGCACGTGTC AGATATATAC ATCCTGT

ACAGGG GGCGCAAA AT TGGCGGTTCC CCTAATGAGG GATCAGAGGT CCGTGCACAC TCTATATATG TAGGACA TGCCTAAGAC /5Cy3/ – 5’

**601-R39-RH**

5’- /5Cy5/ACGGATTCTG TGTCCC CCGCGTT/iBiodT/TA ACCGCCAAGG GGATTACTCC CTAGTCTCCA GGCACGTGTC AGATATATAC ATCCTGT

ACAGGG GGCGCAAA AT TGGCGGTTCC CCTAATGACG GATCAGAGGT CCGTGCACAG TCTATATATG TAGGACA TGCCTAAGAC /5Cy3/ – 5’

**601-R56-RH**

5’- /5Cy5/ACGGATTCTG TGTCCC CCGCGTT/iBiodT/TA ACCGCCAAGG GGATTACTCC CTAGTCTCCA GGCACGTGTC AGATATATAC ATCCTGT

ACAGGG GGCGCAAA AT TCGCGGTTCC CCTAATGAGG GATCAGAGGT CCGTGCACAG TCTATATATG TAGGACA TGCCTAAGAC /5Cy3/ - 5’

**601-RH-16**

5’- /5Cy5/ACGGATTCTG TGTCCC CCGCGTTTTA ACCGCCAAGG GGA/iBiodT/TACTCC CTAGTCTCCA GGCACGTGTC AGATATATAC ATCCTGT

ACAGGG GGCGCAAAAT TGGCGGTTCC CCTA ATGAGG GATCAGAGGT CCGTGCACAG TCTATATATG TAGGACA TGCCTAAGAC /5Cy3/ – 5’

**601-R18-RH-16**

5’- /5Cy5/ACGGATTCTG TGTCCC CCGCGTTTTA ACCGCCAAGG GGA/iBiodT/TACTCC CTAGTCTCCA GGCACGTGTC AGATATATAC ATCCTGT

ACAGGG GGCGCAAAAT TGGCGGTTCC CCTA ATGAGG GATCAGAGGT CCGTGCACAC TCTATATATG TAGGACA TGCCTAAGAC /5Cy3/ – 5’

**R40-RH-TT**

5’- /5Cy5/ACGGATTCTG TGTCCC CCGCGTT/iBiodT/TA ACCGCCAAGG GGATTACTCC CTAGTCTCCA GGCACGTGTC AGATATATAC ATCCTGT

ACAGGG GGCGCAAA AT TGGCGGTTCC CCTAATGTGG GATCAGAGGT CCGTGCACAG TCTATATATG TAGGACA TGCCTAAGAC /5Cy3/ – 5’

**R40-RH-AA**

5’- /5Cy5/ACGGATTCTG TGTCCC CCGCGTT/iBiodT/TA ACCGCCAAGG GGATTACACC CTAGTCTCCA GGCACGTGTC AGATATATAC ATCCTGT

ACAGGG GGCGCAAA AT TGGCGGTTCC CCTAATGAGG GATCAGAGGT CCGTGCACAG TCTATATATG TAGGACA TGCCTAAGAC /5Cy3/ – 5’

**R39-RH-CT**

5’- /5Cy5/ACGGATTCTG TGTCCC CCGCGTT/iBiodT/TA ACCGCCAAGG GGATTACTCC CTAGTCTCCA GGCACGTGTC AGATATATAC ATCCTGT

ACAGGG GGCGCAAA AT TGGCGGTTCC CCTAATGATG GATCAGAGGT CCGTGCACAG TCTATATATG TAGGACA TGCCTAAGAC /5Cy3/ – 5’

**R39-RH-CA**

5’- /5Cy5/ACGGATTCTG TGTCCC CCGCGTT/iBiodT/TA ACCGCCAAGG GGATTACTCC CTAGTCTCCA GGCACGTGTC AGATATATAC ATCCTGT

ACAGGG GGCGCAAA AT TGGCGGTTCC CCTAATGAAG GATCAGAGGT CCGTGCACAG TCTATATATG TAGGACA TGCCTAAGAC /5Cy3/ – 5’

**R41-RH-GG**

5’- /5Cy5/ACGGATTCTG TGTCCC CCGCGTT/iBiodT/TA ACCGCCAAGG GGATTAGTCC CTAGTCTCCA GGCACGTGTC AGATATATAC ATCCTGT

ACAGGG GGCGCAAA AT TGGCGGTTCC CCTAATGAGG GATCAGAGGT CCGTGCACAG TCTATATATG TAGGACA TGCCTAAGAC /5Cy3/ – 5’

**R41-RH-GA**

5’- /5Cy5/ACGGATTCTG TGTCCC CCGCGTT/iBiodT/TA ACCGCCAAGG GGATTAATCC CTAGTCTCCA GGCACGTGTC AGATATATAC ATCCTGT

ACAGGG GGCGCAAA AT TGGCGGTTCC CCTAATGAGG GATCAGAGGT CCGTGCACAG TCTATATATG TAGGACA TGCCTAAGAC /5Cy3/ – 5’

**R41-RH-GT**

5’- /5Cy5/ACGGATTCTG TGTCCC CCGCGTT/iBiodT/TA ACCGCCAAGG GGATTATTCC CTAGTCTCCA GGCACGTGTC AGATATATAC ATCCTGT

ACAGGG GGCGCAAA AT TGGCGGTTCC CCTAATGAGG GATCAGAGGT CCGTGCACAG TCTATATATG TAGGACA TGCCTAAGAC /5Cy3/ – 5’

**References**

1. Ngo, T.T., Zhang, Q., Zhou, R., Yodh, J.G. & Ha, T. Asymmetric unwrapping of nucleosomes under tension directed by DNA local flexibility. *Cell* **160**, 1135-44 (2015).
